# Association between micronutrients and myopia in American adolescents: evidence from the 2003–2006 National Health and Nutrition Examination Survey

**DOI:** 10.3389/fnut.2024.1477403

**Published:** 2024-10-03

**Authors:** Kunhong Xiao, Ruiye Chen, Rong Lin, Wenyi Hu, Jiahao Liu, Mayinuer Yusufu, Yan Huang, Li Li

**Affiliations:** ^1^Shengli Clinical Medical College of Fujian Medical University, Fujian Provincial Hospital, Fuzhou University Affiliated Provincial Hospital, Fuzhou, China; ^2^Department of Ophthalmology and Optometry, Fujian Medical University, Fuzhou, China; ^3^Centre for Eye Research Australia, Royal Victorian Eye and Ear Hospital, East Melbourne, VIC, Australia; ^4^Department of Surgery (Ophthalmology), The University of Melbourne, Melbourne, VIC, Australia; ^5^Department of Ophthalmology, West China Xiamen Hospital of Sichuan University, Xiamen, China

**Keywords:** micronutrients, myopia, NHANES, Cis-*β*-carotene, diet

## Abstract

**Purpose:**

To investigate the associations between circulating micronutrients (vitamins A, C, D, E, and carotenoids) and the risk of myopia.

**Methods:**

A total of 1,620 adolescents from the 2003–2006 National Health and Nutrition Examination Survey (NHANES) were included. Logistic regression was used to analyze the associations of micronutrients with myopia and high myopia. Restricted cubic spline analysis was employed to assess the potential nonlinear relationships.

**Results:**

Among the 1,620 adolescents, 549 were diagnosed with myopia. After adjusting for multiple covariates, only cis-*β*-carotene was significantly associated with the risk of myopia (OR 1.19, 95% CI 1.03–1.39) and high myopia (OR 1.44, 95% CI 1.03–2.03). No significant associations were found between vitamins A, D, E, C, *α*-carotene, trans-*β*-carotene, lutein zeaxanthin, and myopia. No nonlinear relationships were observed between any of the micronutrients and myopia.

**Conclusion:**

Cis-β-carotene is significantly associated with an increased risk of myopia and high myopia. Further research is needed to understand the underlying mechanisms and potential impact of cis-*β*-carotene on ocular health.

## 1 Introduction

Myopia has become a significant public health issue among adolescents. In parts of East and Southeast Asia, the prevalence of myopia among high school students is estimated to be around 80 to 90% (1). High myopia can lead to pathological conditions such as retinal detachment, glaucoma, and myopic maculopathy, which may result in irreversible vision loss (2). Given the increasing incidence and potential for severe ocular complications, it is crucial to understand the factors contributing to the development and progression of myopia.

Recent studies have highlighted the role of various micronutrients in maintaining eye health (3, 4). Micronutrients, including vitamins and minerals, are essential for many physiological functions in the eye. For instance, vitamin A is vital for maintaining normal vision and preventing night blindness (5). High-dose vitamin C and E supplements may delay the progression of age-related macular degeneration and improve vision (6). Carotenoids, by reducing reactive oxygen species, inhibiting inflammation, and suppressing inflammatory markers, have shown significant preventive and therapeutic benefits for age-related ocular abnormalities (7).

Despite the well-recognized importance of micronutrients in eye health, their relationship with myopia remains unclear. Previous studies investigating the association between vitamin D and myopia have produced contradictory results. Yazar et al. (8) found that individuals with vitamin D deficiency have a significantly higher rate of myopia compared with those with sufficient vitamin D levels, while Williams et al. (9) reported no significant association between vitamin D and the risk of myopia. Additionally, evidence on the effects of other micronutrients on myopia is relatively limited.

This study aims to investigate the associations between various serum micronutrient levels (vitamins A, C, D, E, *α*-carotene, trans-*β*-carotene, cis-β-carotene, lutein, and zeaxanthin) and myopia among adolescents in the United States, using data from the 2003–2006 National Health and Nutrition Examination Survey (NHANES). By examining this association, we seek to identify specific micronutrients that may influence the risk of myopia. Understanding these relationships could provide insights into potential nutritional interventions to prevent or slow the progression of myopia, ultimately contributing to improved eye health.

## 2 Methods

### 2.1 Data source and study population

We used data from the NHANES database, a continuous series of cross-sectional surveys conducted biennially by the National Center for Health Statistics (NCHS) of the Centers for Disease Control and Prevention. Details on NHANES data collection can be found at the NCHS (10). We utilized data from two independent NHANES cycles (2003–2004 and 2005–2006). The inclusion criteria for our study population were adolescents aged 12–19 years at each recruitment cycle. Each cycle is considered a separate, independent population. Exclusion criteria included: (1) subjects lacking exposure variables, i.e., serum micronutrient levels; (2) subjects without available refractive error data; and (3) subjects missing data on covariates such as age, sex, race, education level, poverty index, height, weight, and mean total cholesterol. All subjects aged 12 years and older underwent examinations at the Mobile Examination Center (MEC). Refractive error was assessed using an automated refraction device. Myopia was defined as a spherical equivalent (SE) of ≤ −1.0 diopters (D) in at least one eye (11). Adolescents with SE ≤ −6.00 D were classified as having high myopia.

### 2.2 Micronutrients assessment

The assessment of serum micronutrients has been detailed in previous studies (12). Blood samples from participants were collected at the Mobile Examination Center (MEC) and transported to designated laboratories. The serum levels of vitamins A, C, E, and carotenoids were measured using high-performance liquid chromatography and multi-wavelength photodiode array absorbance detection. The serum concentration of vitamin D was measured using the DiaSorin RIA kit (Detailed information about these measurement methods can be found at NHANES Lab Methods). According to previous studies, deficiencies in vitamins A, C, D, and E are defined as less than 0.7 μmol/L (13), 11.4 μmol/L (14), 50 nmol/L (15), and 9 μmol/L (16), respectively. Deficiencies in serum *α*-carotene, trans-*β*-carotene, and lutein/zeaxanthin are defined as less than 0.836 μg/dL (12), 4.12 μg/dL (17), and 7.23 μg/dL (6), respectively. Due to the lower quartile of serum cis-β-carotene being below the detection limit, cis-β-carotene deficiency was not analyzed in this study.

### 2.3 Covariate assessment

Demographic and socioeconomic data, including age, sex, race/ethnicity (non-Hispanic White, non-Hispanic Black, Mexican American, or Other), education level (less than high school, high school diploma or above), and poverty income ratio (PIR < 1.0, PIR ≥ 1.0), were obtained from interviews. Weight, height, and total cholesterol were measured either at the MEC or in participants’ homes. Overweight was defined as a body mass index (BMI) of >25 kg/m^2^. Considering that fat-soluble vitamins (such as vitamins A, D, and E) and carotenoids (such as *β*-carotene) require cholesterol for metabolism and transport, controlling for total cholesterol levels helps reduce potential confounding effects on the relationship between fat-soluble nutrients and myopia.

### 2.4 Statistical analysis

All analyses were conducted using the statistical software R version 4.4.1, employing NHANES clustering design variables (SDMVSTRA, SDMVPSU) and the full sample 2-year MEC exam weights (WTMEC2YR) for the two cycles (2003–2004, 2005–2006). Weighted methods were used to analyze the associations between demographic factors, myopia, and micronutrients. Continuous data were summarized using means and quartiles, while categorical data were reported using unweighted counts and weighted percentages. Comparisons between subjects with and without myopia employed t-tests for continuous data and design-adjusted Rao-Scott Pearson χ^2^ tests for categorical data. A two-sided *p*-value <0.05 was considered statistically significant. We performed Z-standardization on serum micronutrient levels to calculate the odds ratios (ORs) and 95% confidence intervals (CI) for each 1 standard deviation (SD) increase.

Logistic regression was used to explore the association between each micronutrient and myopia among the subjects. Model 1 was adopted a univariate analysis. Model 2 was adjusted for age, sex, race PIR, education, weight, and height. Model 3 additionally adjusted for serum total cholesterol to account for the presence of fat-soluble micronutrients (vitamins A, D, E, and carotenoids). Furthermore, the relationship between high myopia and micronutrients was considered. Restricted cubic splines were used to examine the nonlinear associations, with the analysis performed at 4 knots.

## 3 Results

### 3.1 Basic characteristics of the study population

**Figure 1:**
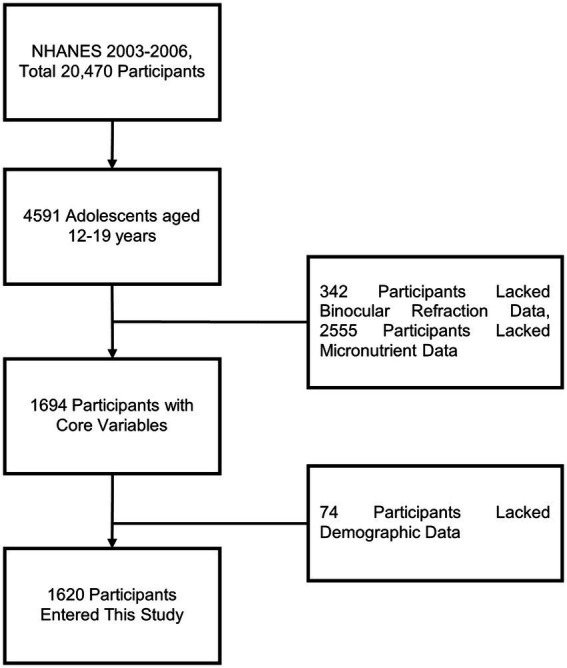
Flowchart describing the research process for exploring the association between micronutrients and myopia.

From 2003 to 2006, a total of 20,470 participants were included in the NHANES study, of which 4,591 were adolescents aged 12–19 years. After excluding 2,971 participants due to missing core variables and covariates, 1,620 participants were included in the final analysis (Figure 1).

In this study, among the 1,620 participants, 32% (549 individuals, 36 of whom had high myopia) were classified into the myopia group, while 68% (1,071 individuals) were classified into the non-myopia group. As shown in Table 1, the median age of the overall population was 15 years [interquartile range (IQR) 14 to 17 years], with an almost equal gender distribution (48% female and 52% male). The racial distribution was as follows: 12% Mexican American, 14% non-Hispanic Black, 64% non-Hispanic White, 6% Multiracial, and 3.9% other Hispanic. The median weight and height were 62 kg (IQR 53 to 76 kg) and 166 cm (IQR 159 to 174 cm), respectively. Participants with myopia tended to be taller (*p* = 0.018).

**Table 1:** Characteristics of participants without and with myopia in the 2003–2006 National Health and Nutrition Examination Survey.

Characteristic	Overall, *N* = 1,620 (100%)	Myopia, *N* = 549 (32%)	Non-myopia, *N* = 1,071 (68%)	*p*- value
Age	15.00 (14.00, 17.00)	16.00 (14.00, 17.00)	15.00 (13.00, 17.00)	0.3
Sex				0.7
Female	811 (48%)	271 (47%)	540 (49%)	
Male	809 (52%)	278 (53%)	531 (51%)	
Race				0.051
Mexican American	535 (12%)	187 (13%)	348 (11%)	
Non-Hispanic Black	529 (14%)	183 (15%)	346 (14%)	
Non-Hispanic White	434 (64%)	131 (59%)	303 (67%)	
Other/Multiracial	79 (6.0%)	31 (6.8%)	48 (5.6%)	
Other Hispanic	43 (3.9%)	17 (6.5%)	26 (2.7%)	
PIR				0.3
At or above poverty line	1,130.00 (80.98%)	395.00 (82.67%)	735.00 (80.17%)	
Below poverty line	490.00 (19.02%)	154.00 (17.33%)	336.00 (19.83%)	
Education				0.10
Below high school	1,344 (84%)	444 (81%)	900 (85%)	
High school or higher	276 (16%)	105 (19%)	171 (15%)	
Weight (Kg)	62 (53, 76)	63 (54, 77)	62 (53, 73)	0.2
Height (cm)	166 (159, 174)	167 (159, 176)	165 (158, 173)	**0.018**
Mean total cholesterol (mg/dL)	157 (137, 176)	156 (135, 178)	158 (140, 175)	0.4
Vitamin A (umol/L)	1.60 (1.38, 1.87)	1.65 (1.41, 1.88)	1.57 (1.37, 1.86)	0.086
Vitamin E(umol/L)	17.54 (15.21, 20.41)	17.65 (14.93, 20.69)	17.51 (15.31, 20.39)	0.6
Cis-β-carotene(umol/L)	0.009 (0.009, 0.015)	0.009 (0.009, 0.015)	0.009 (0.009, 0.015)	0.081
Alpha-carotene (ug/dL)	1.80 (1.00, 3.10)	1.80 (0.90, 3.40)	1.70 (1.00, 3.00)	0.4
Trans-Beta carotene (ug/dL)	9.10 (6.10, 14.10)	9.20 (6.20, 14.50)	8.90 (6.00, 13.80)	0.12
Lutein and zeaxanthin (ug/dL)	10.62 (8.10, 13.80)	10.83 (8.17, 14.40)	10.54 (8.10, 13.50)	0.4
Vitamin D(nmol/L)	61.60 (47.10, 73.80)	61.60 (47.10, 76.20)	61.60 (49.50, 73.80)	0.8
Vitamin C(nmol/L)	60.80 (43.70, 74.90)	60.67 (43.20, 75.50)	60.80 (43.70, 74.40)	0.8
Vitamin A deficiency				0.5
Not	1,619 (100%)	549 (100%)	1,070 (100%)	
Yes	1 (<0.1%)	0 (0%)	1 (<0.1%)	
Vitamin C deficiency				0.6
Not	1,596 (97%)	543 (97%)	1,053 (98%)	
Yes	24 (2.6%)	6 (3.0%)	18 (2.4%)	
Vitamin D deficiency				0.3
Not	821 (70%)	265 (68%)	556 (71%)	
Yes	799 (30%)	284 (32%)	515 (29%)	
Vitamin E deficiency				0.2
Not	1,616 (100%)	548 (99%)	1,068 (100%)	
Yes	4 (0.3%)	1 (0.6%)	3 (0.1%)	
α-carotene deficiency				0.6
Not	1,458 (90%)	492 (91%)	966 (90%)	
Yes	162 (10.0%)	57 (9.2%)	105 (10%)	
Trans-β-carotene deficiency				0.2
Not	1,477 (91%)	506 (93%)	971 (91%)	
Yes	143 (8.7%)	43 (7.2%)	100 (9.3%)	
Lutein and zeaxanthin deficiency				0.2
Not	1,407 (81%)	482 (83%)	925 (79%)	
Yes	213 (19%)	67 (17%)	146 (21%)	

Bold values indicate *p* < 0.05.

### 3.2 Micronutrients and myopia

**Figure 2:**
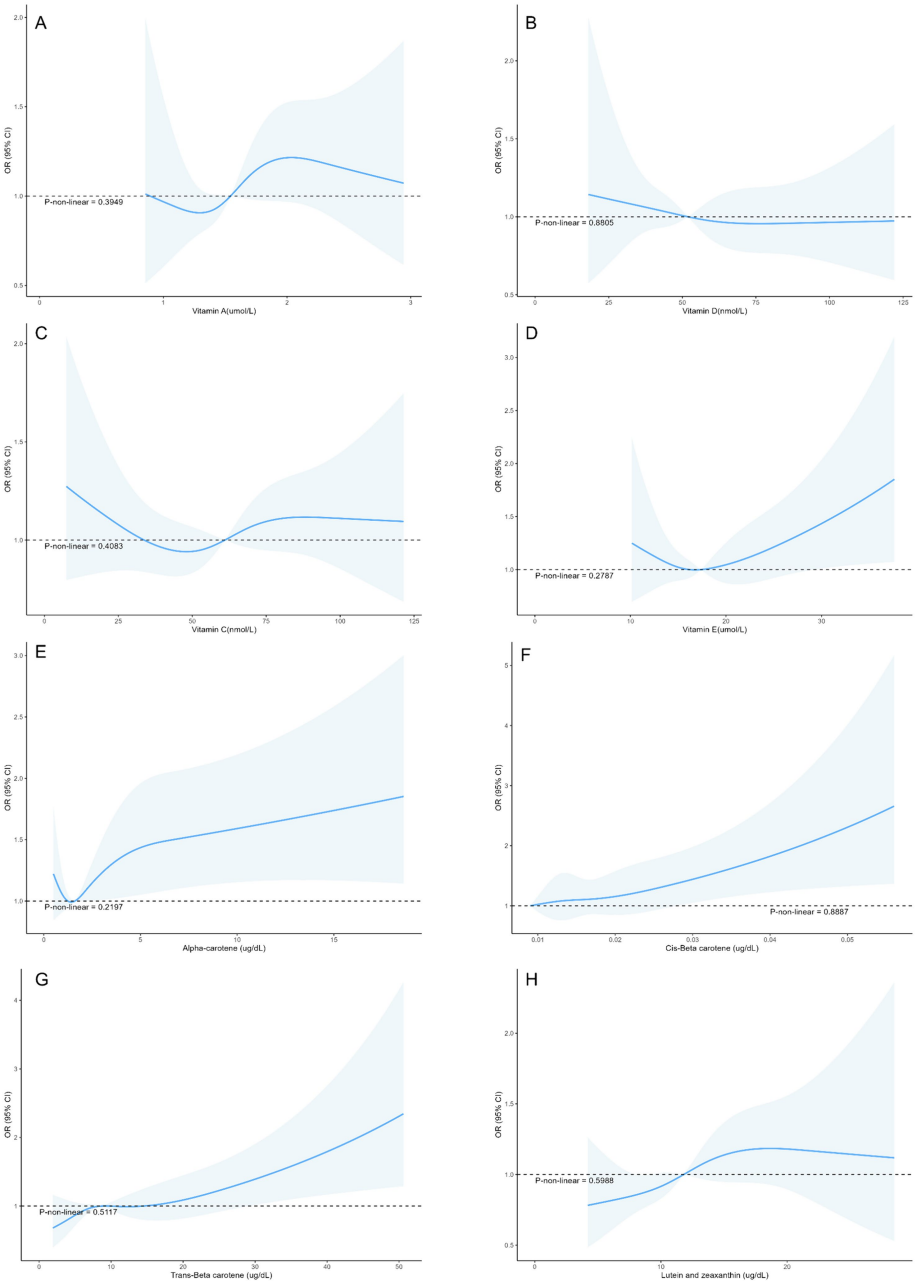
Restricted cubic splines between micronutrient levels and the risk of myopia in adolescents **(A)** Vitamin A, **(B)** Vitamin D, **(C)** Vitamin C, **(D)** Vitamin E, **(E)** Alpha-carotene, **(F)** Cis-*β*-carotene, **(G)** Trans-β-carotene, **(H)** Lutein and zeaxanthin.

The associations between micronutrients and myopia based on data from the 2003–2006 NHANES are shown in Table 2. Cis-*β*-carotene was significantly associated with myopia (Model 3: OR = 1.19, 95% CI: 1.03–1.39, *p* = 0.026). Trans-β-carotene showed a significant association in Model 1 (OR = 1.17, 95% CI: 1.01–1.34, *p* = 0.036) but the result did not remain significance in Models 2 and 3. Other micronutrients such as vitamins A, D, E, C, *α*-carotene, lutein and zeaxanthin were not statistically significantly associated with myopia in the models. Additionally, no nonlinear associations were found in the restricted cubic spline analysis (Figure 2).

**Table 2:** Association between micronutrients and myopia in the 2003–2006 National Health and Nutrition Examination Survey.

Micronutrients	Model 1			Model 2			Model 3		
	OR	95% CI	*p*- value	OR	95% CI	*p*- value	OR	95% CI	*p*- value
Vitamin D, per SD	1.00	0.91, 1.10	>0.9	0.96	0.87, 1.06	0.4	0.96	0.86, 1.07	0.4
Vitamin A, per SD	1.11	0.96, 1.28	0.14	1.08	0.92, 1.27	0.3	1.12	0.97, 1.30	0.11
Vitamin E, per SD	1.03	0.87, 1.21	0.7	0.98	0.78, 1.24	0.9	1.08	0.85, 1.36	0.5
Vitamin C, per SD	1.00	0.89, 1.12	>0.9	0.99	0.85, 1.14	0.8	0.99	0.85, 1.14	0.8
α-carotene, per SD	1.12	0.95, 1.31	0.2	0.99	0.75, 1.31	>0.9	0.99	0.73, 1.32	>0.9
Trans-β-carotene, per SD	1.17	1.01, 1.34	**0.036**	1.14	0.60, 2.15	0.7	1.17	0.62, 2.22	0.6
Lutein and zeaxanthin, per SD	1.06	0.92, 1.22	0.4	1.02	0.84, 1.24	0.8	1.06	0.88, 1.28	0.5
Cis-β-carotene, per SD	1.17	1.01, 1.36	**0.040**	1.18	1.03, 1.36	**0.023**	1.19	1.03, 1.39	**0.026**

Bold values indicate *p* < 0.05.Model 1: Univariate analysis; Model 2: adjusted for age, sex, race, PIR, education, weight and height; Model 3: adjusted for age, sex, race, PIR, education, weight, height and mean total cholesterol.

### 3.3 Micronutrients and high myopia

The associations between micronutrients and high myopia based on data from the 2003–2006 NHANES are presented in Table 3.Cis-*β*-carotene showed a significant association with high myopia (Model 3: OR = 1.44, 95% CI: 1.03–2.03, *p* = 0.038). Other micronutrients did not show statistically significant associations with high myopia.

**Table 3:** Association between micronutrients and high myopia in the 2003–2006 National Health and Nutrition Examination Survey.

Characteristic	Model 1			Model 2			Model 3		
	OR	95% CI	*p*- value	OR	95% CI	*p*- value	OR	95% CI	*p*- value
Vitamin D, per SD	0.98	0.54, 1.80	>0.9	0.95	0.47, 1.93	0.9	0.91	0.43, 1.92	0.8
Vitamin A, per SD	1.09	0.70, 1.69	0.7	1.14	0.65, 2.02	0.6	1.06	0.52, 2.18	0.8
Vitamin E, per SD	1.26	0.92, 1.73	0.13	1.19	0.82, 1.74	0.3	1.22	0.69, 2.14	0.4
Vitamin C, per SD	1.24	0.79, 1.95	0.3	1.09	0.66, 1.80	0.7	1.08	0.64, 1.83	0.7
α-carotene, per SD	1.44	1.16, 1.77	**0.002**	1.15	0.63, 2.12	0.6	1.18	0.61, 2.26	0.6
Trans-β-carotene, per SD	1.39	1.03, 1.87	**0.034**	0.53	0.13, 2.08	0.3	0.49	0.12, 2.01	0.3
Lutein and zeaxanthin, per SD	1.06	0.73, 1.53	0.7	0.75	0.44, 1.28	0.3	0.68	0.37, 1.25	0.2
Cis-β-carotene, per SD	1.47	1.07, 2.01	**0.020**	1.43	1.04, 1.96	**0.032**	1.44	1.03, 2.03	**0.038**

Bold values indicate *p* < 0.05.Model 1: Univariate analysis; Model 2: adjusted for age, sex, race, PIR, education, weight and height; Model 3: adjusted for age, sex, race, PIR, education, weight, height and mean total cholesterol.

## 4 Discussion

The prevalence of myopia among school-aged children in North America is reported to be 42% (18). Parents of children with myopia often seek dietary advice from ophthalmologists (19), but research in this area remains relatively limited. This study systematically evaluated the association between various micronutrients and myopia among adolescents based on data from the NHANES from 2003 to 2006. A total of 1,620 participants were included, with a myopia prevalence of 32% among adolescents aged 12–19 years. Our results indicated that higher serum cis-*β*-carotene levels were associated with an increased risk of myopia and high myopia in adolescents. Other micronutrients, such as vitamins A, D, E, C, *α*-carotene, trans-*β*-carotene, lutein, and zeaxanthin, were not statistically significantly associated with adolescent myopia.

To the best of our knowledge, this is the first report linking higher serum cis-*β*-carotene levels with an increased risk of myopia. Moreover, since β-carotene is an exogenous rather than endogenous antioxidant (18), caution should be exercised when considering β-carotene supplements for young people. While past research indicated that cis-β-carotene is beneficial for the retina (20, 21), some studies have found adverse effects. One year of treatment with *Dunaliella* containing cis-β-carotene adversely affects full-field electroretinography (ERG) amplitudes in patients with RDH5-related fundus albipunctatus and leads to damage to both cone and rod cells (22). The authors suggested that this may be related to the increased rate of 11-cis retinal photoisomerization, leading to elevated A2E accumulation (22, 23). Interestingly, the accelerated biosynthesis of A2E and its conversion to epoxides have been shown to potentially contribute to myopia (24), which may partially explain our findings. Moreover, although *β*-carotene as an antioxidant may theoretically protect the retina by reducing oxidative stress (25, 26), high doses of β-carotene supplements were reported to have strong side effects, including mitochondrial dysfunction and increased oxidative stress, negatively impacting retinal cells (27). The National Institutes of Health (NIH) Office of Dietary Supplements also advises that *β*-carotene supplements are not recommended for the general population (28). The existing evidence aligns with our finding that cis-*β*-carotene intake should be carefully controlled in dietary supplements, especially for adolescents aged 12–19 years.

Furthermore, in this study, only cis-β-carotene was significantly positively associated with myopia risk, while trans-*β*-carotene did not show a significant association. Previous studies have indicated that the energy barrier for reverse cis-to-trans isomerization is lower than that for direct isomerization, allowing cis isomers of carotenoids to react more rapidly with free radicals (29, 30). Additionally, *β*-carotene can be metabolized into retinol in the retinal pigment epithelium (RPE) cells and further converted into rhodopsin (31). We hypothesize that the accumulation of cis-β-carotene may lead to increased local oxidative stress, resulting in structural and functional changes in the retina, thereby promoting the development of myopia. Further research is needed to explore the specific mechanisms of cis-β-carotene in the retina, particularly its effects on RPE cells and receptors, and how these effects are related to the pathogenesis of myopia.

Vitamin A is involved in the formation of rhodopsin and the conversion of light signals (32). However, Fletcher et al. (33) proposed that a high intake of vitamin A during adolescence does not necessarily reduce the risk of myopia in early adulthood, which is consistent with our study. Additionally, in the RCS curve of serum vitamin A and myopia, the wider confidence interval may be due to the lack of samples with high vitamin A concentrations. This suggests the need for further research to verify their nonlinear relationship. Similarly, *α*-carotene, a precursor of vitamin A, can also convert into retinal and participate in rhodopsin formation (34). Previous studies have pointed out that the expression of rhodopsin has a relatively limited impact on defocus myopia (35). The impact of vitamin D on myopia remains controversial. Some scholars believe that low blood levels of vitamin D are associated with an increased risk of myopia (8). However, some studies suggest that the contribution of vitamin D levels to myopia is ignorable, with previous results likely confounded by sun exposure during outdoor activity time (36, 37). Therefore, further studies need to control for these confounding factors to more accurately assess the relationship between vitamin D and myopia. Vitamins C and E can prevent oxidative stress-induced cellular damage and help reduce ocular inflammation. In the Age-Related Eye Disease Study (AREDS), supplementation with vitamins C and E was found to reduce the risk of cataracts and glaucoma, among other eye diseases (38). However, Zheng et al. (39) found that in a sample of American adults, vitamin E levels were not associated with an increased or decreased risk of myopia, which is consistent with our study. Furthermore, a study in Hong Kong compared the vitamin C intake of 24 children who developed myopia between the ages of 7 and 10 with that of 68 children who did not develop myopia by the age of 10, and found statistically significant differences (40). This contradicts our findings. On one hand, vitamin C intake may not accurately reflect its bioavailability in the body. On the other hand, the sample size limits the reliability of the study. However, further randomized controlled trials (RCTs) are necessary to validate the true effects of micronutrients on myopia.

This study used a nationally representative sample and comprehensively evaluated the relationship between circulating micronutrients and myopia. However, there are several limitations. First, given the cross-sectional design of the NHANES data, we were not able to infer the longitudinal relationship of micronutrients and future risk of myopia. Secondly, due to the lack of data on outdoor activity time and near-work time in NHANES, our study inevitably has residual confounding. Future longitudinal studies, RCTs, and biological research are needed to provide a more comprehensive analysis and accurate conclusions. Lastly, the majority of NHANES participants are Non-Hispanic White, which may limit the generalizability of the results in other populations.

## 5 Conclusion

In summary, this population-based study found that higher serum cis-*β*-carotene levels were associated with an increased risk of myopia and high myopia in adolescents, indicating that cis-β-carotene is a risk factor for myopia in US adolescents. These findings suggest potential dietary guidance for myopia prevention. However, further research is needed to understand the underlying mechanisms and potential impact of cis-β-carotene on ocular health. Comprehensive evaluation through RCTs is recommended to fully assess the effects of micronutrients on adolescent myopia.

## Data Availability

The original contributions presented in the study are included in the article/supplementary material, further inquiries can be directed to the corresponding authors.
